# Topographical functional connectivity patterns exist in the congenitally, prelingually deaf

**DOI:** 10.1038/srep29375

**Published:** 2016-07-18

**Authors:** Ella Striem-Amit, Jorge Almeida, Mario Belledonne, Quanjing Chen, Yuxing Fang, Zaizhu Han, Alfonso Caramazza, Yanchao Bi

**Affiliations:** 1 Department of Psychology, Harvard University, Cambridge, MA 02138, USA; 2 Faculty of Psychology and Educational Sciences, University of Coimbra, Coimbra 3001-802, Portugal; 3 Proaction Laboratory, Faculty of Psychology and Educational Sciences, University of Coimbra, Coimbra 3001-802, Portugal; 4 State Key Laboratory of Cognitive Neuroscience and Learning & IDG/McGovern Institute for Brain Research, Beijing Normal University, Beijing 100875, China; 5 Center for Mind/Brain Sciences, University of Trento, 38068, Rovereto, Italy

## Abstract

Congenital deafness causes large changes in the auditory cortex structure and function, such that without early childhood cochlear-implant, profoundly deaf children do not develop intact, high-level, auditory functions. But how is auditory cortex organization affected by congenital, prelingual, and long standing deafness? Does the large-scale topographical organization of the auditory cortex develop in people deaf from birth? And is it retained despite cross-modal plasticity? We identified, using fMRI, topographic tonotopy-based functional connectivity (FC) structure in humans in the core auditory cortex, its extending tonotopic gradients in the belt and even beyond that. These regions show similar FC structure in the congenitally deaf throughout the auditory cortex, including in the language areas. The topographic FC pattern can be identified reliably in the vast majority of the deaf, at the single subject level, despite the absence of hearing-aid use and poor oral language skills. These findings suggest that large-scale tonotopic-based FC does not require sensory experience to develop, and is retained despite life-long auditory deprivation and cross-modal plasticity. Furthermore, as the topographic FC is retained to varying degrees among the deaf subjects, it may serve to predict the potential for auditory rehabilitation using cochlear implants in individual subjects.

Audition is an important sensory modality for communication. Hearing impairment, a relatively frequent condition^1^, results in significant limitation to everyday life. In the recent decades cochlear implants (CI) have developed to a level that they can be used to alleviate even congenital or early-age profound hearing loss. However, CI success is restricted by brain developmental critical periods^2^,^3^, in that it has a limited time-window for its application. If implantation does not occur very early in life, deaf children do not successfully develop complex auditory skills, and suffer from impaired language processing^4^,^5^,^6^.

This malfunction appears to depend on two factors: the recruitment of the auditory cortex, in the absence of auditory input, for non-auditory functions, such as visual motion processing and sign language^7^,^8^,^9^,^10^,^11^,^12^,^13^,^14^,^15^,^16^,^17^,^18^,^19^,^20^,^21^,^22^, and the dependence of auditory system development on intact sensory auditory experience during development^2^,^5^,^12^. It was also shown that the level of cross-modal plastic recruitment of the auditory cortex in deaf people can predict how much they may benefit from auditory restoration using cochlear implants^23^,^24^,^25^, creating a direct link between cross-modal plasticity and auditory functional retention. Thus, evaluating the recruitment of the auditory cortex during visual tasks using fMRI may be of prognostic medical value. But what is known about the second factor affecting auditory restoration, the genuine role of the auditory cortex and its retained development in the absence of auditory experience?

Although much in-depth research has been conducted in various animal models of deafness, mainly focusing on subcortical auditory nuclei and pathways (reviewed in refs 2 and 12), and showing mixed effects of cross-modal plasticity of visual and somatosensory origin and retained organization^26^,^27^,^28^,^29^,^30^, the auditory system of humans bears critical differences from that of other mammals, due to the effects of language processing (e.g.^31^). Indeed, processing sign language is one of the important causes of cross-modal plasticity in the auditory cortex of deaf people^19^,^32^,^33^,^34^,^35^,^36^. Despite this important difference, the study of auditory organization in deaf humans is more limited, in part due to methodological difficulties. Magnets are commonly used to attach the CI parts, hindering the use of noninvasive measures of fMRI or MEG functional imaging of auditory activity in individuals with CIs. In the absence of a CI, it is impossible to investigate auditory functional organization since there is no way to (non-invasively) provide auditory stimulation to the deaf. Thus, it has proven difficult to map the auditory cortex of the deaf even with regard to its most basic large-scale property, topographic tonotopic organization, which characterizes most processing levels in the auditory pathways^37^,^38^.

However, in recent years it has become apparent that functional brain organization can also be investigated without external sensory stimulation. Functional connectivity MRI (fcMRI;^39^), based on intrinsic slow (<0.1 Hz) BOLD fluctuations in the absence of a task, shows similar spatial patterns to those evident during performance of a task^40^,^41^,^42^,^43^(though dynamic alterations are also present;^44^,^45^). Furthermore, fcMRI is highly correlated with underlying structural connectivity (a combination of direct and indirect polysynaptic connectivity;^46^,^47^,^48^), and may also be used to study brain topography^49^,^50^,^51^. Importantly, resting-state functional connectivity also correlates with functional and structural anatomy changes due to brain plasticity and usage^52^,^53^, reflecting ongoing developmental changes in network engagement^54^,^55^. Thus, functional connectivity is a sensitive measure to investigate both stable anatomical connectivity as well as plastic changes through life, in normal conditions, atypical development, and disease.

This method allows us to investigate the auditory-based topographic (cochleotopic/tonotopic) network organization of the auditory cortex of the deaf (without CI or auditory experience) even in the absence of auditory stimulation. If auditory experience during postnatal critical periods is necessary for cortex topographical patterns to emerge, or if life-long cross-modal plasticity greatly affects the organization and connectivity patterns of the cortex in the deaf, then topographical fcMRI organization should not be found in the deaf. However, if prenatal factors (which do not necessitate auditory experience) are sufficient for the emergence of tonotopic gradients, and if cross-modal experience throughout life does not cause these to degenerate, then it may be possible to find traces of functional connectivity (FC) replicating tonotopic gradients, even in congenital deafness.

## Results

**Figure 1:**
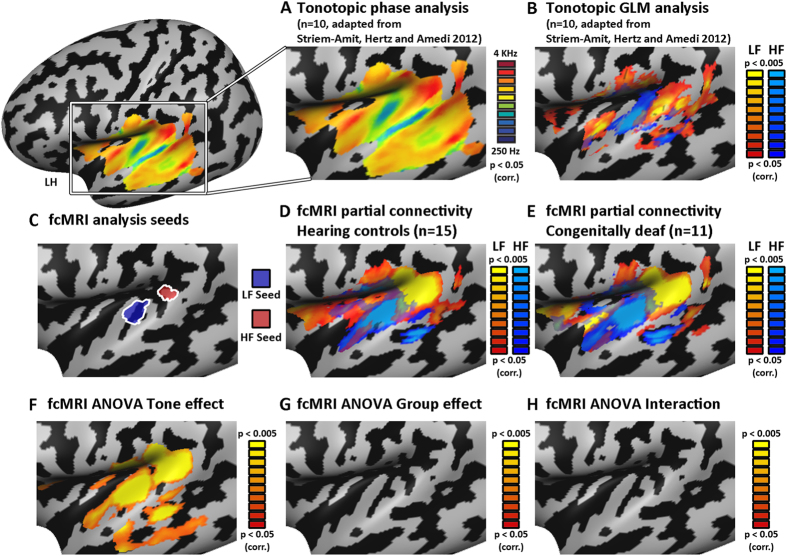
Topographic gradients in fcMRI of the deaf auditory cortex mimic tonotopic organization. (**A)** Tonotopic gradients are presented on a left inflated cortical hemisphere, and cover a large extent of the temporal lobe when mapped directly using auditory stimuli analyzed with phase-encoding analyses in normally hearing subjects (adapted with permission from^56^). (**B)** Tonotopic peaks are identifiable using a direct binary contrast (HF > LF and vice-versa) in task-based tonotopy experiment. Both the core auditory gradient of HF-LF-HF along the superior temporal plane as well as the belt LF lateral band and STG-STS LF peaks are recognizable in this contrast (see peak marking in Figure S1A,B). (**C)** For fcMRI analysis, seeds corresponding to high-frequency (HF; marked red) and low-frequency (LF; marked blue) peaks in the core auditory cortex were chosen. These seeds were used in a partial-correlation mapping minimizing the common components between the two seeds, illuminating differential FC preferences for LF- and HF-preferring regions. (**D)** fcMRI analysis of the hearing control group (random-effect GLM analysis) reveals the topographical organization of functional-connectivity which mimics the tonotopic-organization within the auditory core, belt and beyond. Spatial-similarity-analysis quantitatively supports the significant similarity between the tonotopic-gradients and the FC topographic maps (see results). (**E)** fcMRI analysis (random-effect GLM analysis) reveals topographical organization in the auditory cortex of the congenitally deaf group, which greatly resembles the tonotopic patterns, extending from the core auditory cortex to speech/voice regions and beyond them. For marking of the comparable peaks between FC and tonotopy and across the groups see Figure S1. A spatial-similarity-analysis quantitatively supports the significant similarity between the tonotopic gradients and the FC topographic maps (see results). This suggests these topographical patterns develop regardless of auditory experience. **(F–H)** Analysis of variance (ANOVA) of the two groups shows that while most of the auditory cortex of both groups is selective for one seed over the other (**F**, seed/tone effect; see also MGN; Figure S2), it shows no significant group effect (**G**) or group X seed interaction (**H**), suggesting that the FC large-scale topographical patterns are identical across groups. For effects of group and interaction outside the auditory cortex see Figure S8, Tables S1,2.

**Figure 2:**
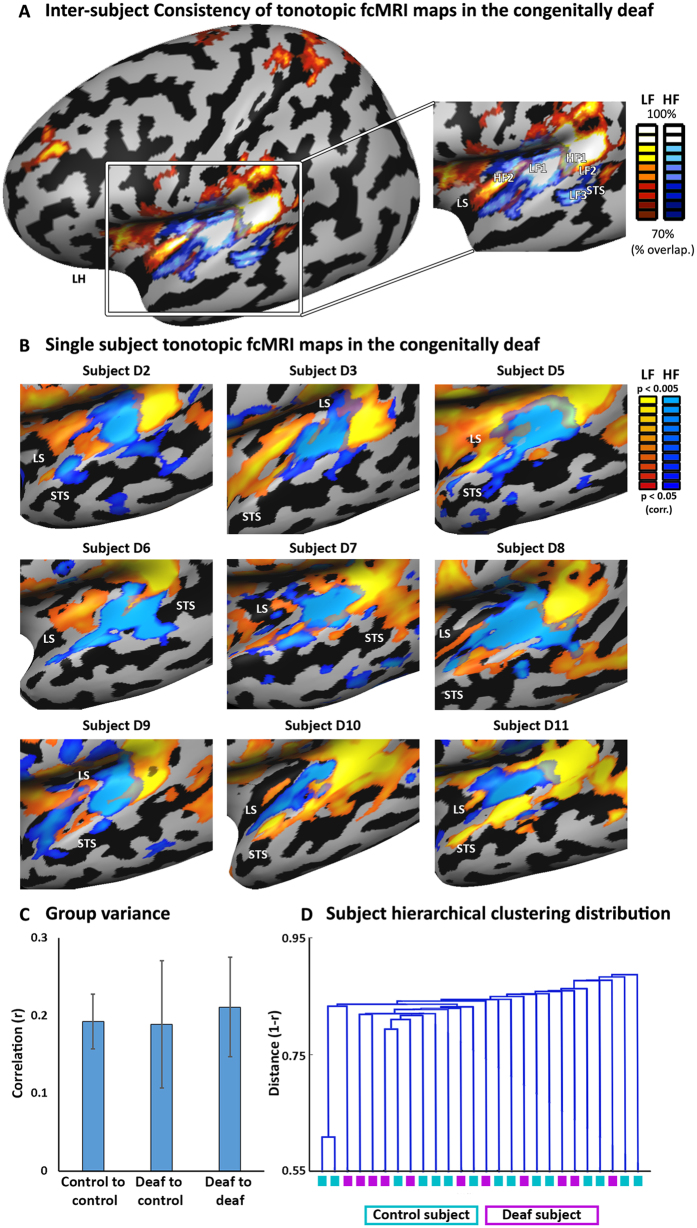
Reproducibility of tonotopic fcMRI maps in the single-subject level. (**A)** All single-subject maps for each of the two seeds (each at p < 0.05, corrected) are overlaid and the probability of each voxel to be activated across the subjects is calculated. The resulting map depicts the high inter-subject consistency of the topographical patterns in the deaf. The deaf FC consistently shows not only the core-belt pattern of HF-LF-HF along the temporal plane, but also the posterior-lateral LF band potentially corresponding to speech/voice sensitive regions^71^,^72^,^73^, as well as the more lateral-inferior LF region in the STG-STS. LS – lateral sulcus, STS – superior temporal sulcus. (**B)** Topographical tonotopic-like fcMRI bands of the core and belt, including those of speech-sensitive regions, can be seen at the single subject level in many of the congenitally deaf individuals. Subjects D2, D3, D5 and D6 have never attempted to use hearing-aids (see Table 1 for all the deaf participant characteristics). (**C)** The correlation of the single-subject maps to the leave-one-out group average showed similar variability of subjects FC patterns within and between groups, suggesting again the absence of significant group differences. (**D)** A dendrogram showing the clustering of the subjects based on their FC pattern correlation distances, as computed by a data-driven independent hierarchical clustering analysis. The deaf and hearing controls are intermixed in the clustering, and no clear group distinction can be made.

Functional connectivity was computed for a-priori defined seed regions-of-interest (ROIs) located in the core auditory cortex (approximately at the location of A1) using a separate phase-encoded task-based tonotopic mapping localizer (Fig. 1A,B;^56^) in a separate group of normally hearing controls (n = 10). ROIs were defined in a low-frequency (LF) peak in posterior medial Heschl’s gyrus and in a high frequency (HF) peak in posterior Heschl’s sulcus (Fig. 1C). These seed ROIs were used to compute partial correlation mapping^57^,^58^, minimizing the common (correlated) components between the two seeds. This maximizes the differentiation between connectivity patterns of areas processing high-frequency tones and those processing low-frequency tones within A1, and enables a focused investigation of the topographical FC pattern, which would, in a normally hearing brain, correspond to tonotopic gradients. We used this approach to conduct several analyses, including group random-effect GLM analyses (Fig. 1; for each group separately and for their comparison), multivariate analysis of group differences, single subject analyses and across-subject consistency (Fig. 2), quantification of the similarity between tonotopic gradients and the FC patterns, as well as a quantification of the variance between the single subjects’ patterns in the deaf group and its similarity to the hearing group.

### Group tonotopic organization fcMRI analysis

Resting-state fcMRI random-effect GLM analysis in the hearing control group replicated to a large extent the large-scale tonotopic preference of the core auditory cortex. The tone preference of the core is comprised of topographical mapping pattern of tone-preference shift from high-frequency tones to low-frequency tones and back along the superior temporal plane with Heschl’s gyrus (HG) located within this mirror-symmetric large scale organization^38^,^56^,^59^,^60^,^61^,^62^,^63^,^64^,^65^,^66^,^67^,^68^,^69^, approximately marking the border between core regions A1 and R^69^. This is exactly the pattern found also in the FC partial correlation, such that an additional FC-HF preference was observed anteriorly to the FC-LF peak (Fig. 1D; for marking of the peaks see Figure S1D; core peaks are marked as HF1, LF1 and HF2). The increased connectivity between the two HF peaks, anterior and posterior to HG was previously shown in diffusion based anatomical tractography analysis^70^, forming the anatomical basis for this fcMRI finding.

Beyond the auditory core, tonotopic gradients are harder to identify using only a binary comparison, even for task-based tonotopic experiments. Specifically, when contrasting HF and LF tones in the original tonotopic experiment used to define the seed ROIs (Fig. 1B;^56^), only a partial pattern of the tonotopic organization can be observed beyond the core. This includes the peak of the lateral LF tone band, possibly reflecting the human homologue of area CL in the macaque (peak LF2 in Figure S1A,B;^56^,^59^,^67^,^69^) or corresponding to speech/voice sensitive regions^71^,^72^,^73^, shown to prefer low-frequency sounds^38^,^73^. Additionally, there is evidence for a HF peak and more dominantly a LF peak in the STG and STS (peaks HF3 and LF3 in Figure S1A,B; potentially reflecting auditory parabelt;^56^). Importantly, evidence for all these peaks can be seen in the fcMRI analysis of the hearing control group (Fig. 1D; Figure S1D), supporting our use of this analysis for the investigation of tonotopic FC organization.

We then investigated if such tonotopic-based FC patterns can also be found in people profoundly deaf since birth. Using the same seeds ROIs as in the hearing group, a similar pattern of FC can be seen, including both core as well as extra-core peaks (Fig. 1E; for marked peaks see Figure S1E). The tonotopic-like FC patterns extended all the way to the belt and parabelt regions, including the language areas such as the auditory word-form area in the left anterior STG^74^ and generally the speech/voice sensitive regions^71^,^72^,^73^. Furthermore, although our current dataset may not be of sufficiently high resolution to reveal gradients within the medial geniculate nucleus (MGN), some MGN FC preference was observed in both the deaf and the hearing (Figure S2A,B), suggesting such patterns can be explored further in the future. The cortical topographical fcMRI patterns were also replicated using cortex-based surface alignment rather than volume alignment (see Figure S3; see also single subject data on individual brains; Fig. 2, and unsmoothed data; Figure S4), and applying spatial smoothing only at the surface level (see Figure S5) controlling for any potential spatial coregistration and smoothing artifacts, and confirming the reliability of the findings. Importantly, the observed topographic patterns do not merely depend on spatial local correlations, in which FC changes with the physical distance between the paired areas due to the spatial smoothing and normalization of fMRI data, the spatial properties of the BOLD signal^75^ and generic local and global cortical effects over the cortex^47^,^76^,^77^. This can be observed clearly from the band pattern of the superior temporal plane: as one moves anteriorly from the left hemisphere HF seed, FC first decreases and then increases again (see plot of FC values within and across hemisphere; Figure S6), in a consistent pattern in both hemispheres, according to the typical HF-LF-HF tonotopic organization of these regions in the hearing.

To quantitatively assess the spatial similarity between the tonotopic selectivity map (resulting from the auditory tonotopic localizer) and the FC selectivity map, we computed the concordance correlation coefficient for each group (CCC; see^78^). Significant concordance values were observed for both groups (CCC > 0.23 and CCC > 0.30 for hearing and deaf groups, P < 0.0001 for both), supporting the qualitative similarity between the FC preference and tonotopic organization in both the hearing and the deaf (Fig. 1). Thus, topographical tonotopic-like functional connectivity patterns can arise in the absence of auditory experience.

Does deafness cause any substantial changes in FC-based large-scale topographical maps? We applied several analyses to address this question. In order to investigate the potential differences in FC patterns between the groups at the voxel level, we computed an ANOVA model comprised of a preferred seed (within subject; LF seed and HF seed) effect and a group effect. The preferred seed effect showed that most of the left auditory cortex was indeed FC seed-selective (Fig. 1F; as well as the auditory thalamus; Figure S2C), as would be expected in tonotopic areas. Interestingly, although differences between the groups were evident in other parts of the brain (e.g. right anterior insula, dorsolateral prefrontal cortex, see Figures S7, S8 and Table S1), no group effect or interaction (preferred seed X group) were evident in the auditory cortex (Fig. 1G,H). However, when applying a more permissive correction for multiple comparisons, including only the left temporal auditory cortex, a small cluster showing interaction between seed X group effects was found in the medial auditory cortex (Figure S5H), possibly in the core or medial belt. Additionally, a highly significant interaction was seen in the thalamus at the location of the right medial geniculate nucleus (MGN; see Figure S2D), potentially suggesting some effect of FC plasticity.

We additionally conducted a multivariate classifier searchlight analysis (MVPA) to account for any areas whose internal organization or pattern discriminates deaf from hearing. An fMRI searchlight for brain regions discerning FC maps of deaf and control subjects found no significant clusters within the temporal auditory cortex. Therefore, multivariate analyses did not reveal any group differences in FC-based large-scale topographical maps. To compare the overall pattern of network FC, we quantitatively compared the spatial similarity of the maps of the deaf group to those of the hearing controls, using the CCC measure between the group FC maps. Highly significant concordance values were observed for both seed FC-topographic division maps (CCC > 0.69 and 0.75 for LF and HF seeds, CCC > 0.76 for effect size; contrast maps between LF and HF FC, P < 0.0001 for all). In sum, the auditory tonotopic-like functional connectivity networks of the deaf were comparable, across several analyses, to those of the controls.

### Cross-subject consistency of tonotopic patterns in the deaf

How reliable and consistent are such FC topographic maps in the deaf? To study this, we examined the patterns of organization emerging in single subjects, as well as across all the subjects (Fig. 2). We computed the FC statistical parametric map of the two seeds in each of the subjects and plotted the cross-subject overlap probability map over all the individual subjects in the deaf group (Fig. 2A). The topographic mapping FC gradients of the auditory core and belt were replicated across a large majority of the subjects (Fig. 2A; the probability map minimal threshold is overlap across 7 of the 11 subjects, but in most of the map the overlap is substantially higher).

Importantly, FC-tonotopic-patterned maps were found at the individual deaf participant level in most subjects (Fig. 2B). Intact FC tonotopic-like patterns were found regardless of the etiology of the tested participant (in participants with hereditary or prenatally-acquired deafness; see Table 1) and regardless of the experience with hearing-aid use (only five of the eleven subjects had ever attempted to use hearing aids, but did not find them helpful; see tonotopic-FC maps of four subjects naïve to hearing-aid use in Fig. 2B). However, the topographic-FC patterns were not found in all subjects, and some variability was also seen in the concordance correlation coefficient between the deaf single-subject maps and group map of the controls (CCC values varied between 0.33 and 0.64 in D4 and D11 respectively).

Does the absence of auditory inputs create less consistent maps than those of the controls? The variance of the single subject maps between the groups was also computed by applying a leave-one-out analyses to calculate the correlation between the average map (FC maps, z-normalized effect size; HF-FC minus LF-FC average of n-1 subjects) and each subject, and was found to be similar to the variance within each group (Fig. 2C). Additionally, we conducted a hierarchical clustering analysis of the FC maps, computing a dendrogram of the Euclidean distances between all the maps (Fig. 2D). The deaf and hearing subjects were completely distributed across the distance dendrogram, with the most dissimilar outlier subjects belonging to the control group. Therefore, the data-driven clustering algorithm could not discern the deaf and hearing subjects. These analyses suggest that in addition to the overall similarity in average response in each group, the deaf variability in tonotopically-informed FC maps is well within that of the control subjects.

### Cross-hemispheric FC connectivity

How clear are the FC-tonotopic patterns across the hemispheres? It seems that while the auditory core shows robust and tone-, or seed-specific functional connectivity across the hemispheres, the pattern is less reliable in the belt and parabelt regions. Using LH seeds generates less robust and less extensive overall differential responsivity in the right hemisphere (e.g. as can be seen in the ANOVA seed effect, compare LH, Fig. 1F, and RH, Figure S7C, when using LH seeds). Furthermore, the pattern of tonotopic-like extra-core bands is less clear in the right hemisphere when using LH seeds (Figure S7A). However, this does not seem to differ much between the two groups, as neither a main group effect nor an interaction between group and seed-effect can be found in the temporal auditory cortex in either hemisphere (although some differences are present in the right anterior insula; Figure S7D,E). Furthermore, directly using RH seeds does show a clear FC-tonotopic patterning in the right hemisphere of both groups (Figure S9), confirming that fcMRI can reveal the topographical structure in both groups also for the right hemisphere. Lastly, using each of the seeds (LH and RH) separately (without applying partial correlation analysis) generates largely bilateral activation, with similar strength on both sides. This suggests the laterality emerges from the absence of *selectivity* for different FC seeds rather than an overall absence of bilateral FC, in a pattern that is consistent across groups. This effect supports the findings of a recent FC study in typically hearing subjects, which also showed an interhemispheric decrease of frequency-selective FC, especially with regard to the non-core regions^50^. Therefore, the deaf FC patterns replicate not only the existence of frequency-selective FC of the hearing^50^, but also its decline between the hemispheres.

## Discussion

Our findings suggest that topographic tonotopic-like large-scale functional connectivity patterns can emerge, and are retained through life, in prelingually deaf humans without auditory experience. This is true for the large-scale gradient of the auditory core and belt, and perhaps to some extent also the parabelt, extending to language-related speech/voice sensitive regions^69^,^71^,^72^,^73^ (Fig. 1D,E). The FC maps showed significant spatial similarity to tonotopic organization in both groups. Interestingly, not only the existence of topographic FC organization is retained, but also its hierarchical relative strength, such that it is most robust in the auditory core regions and less so outside the auditory core (replicating a recent FC study in a hearing group;^50^), as well as its hemispheric dissociation – such that FC preference patterns are decreased across the hemispheres^50^, in accordance with their slightly differential functional roles^79^,^80^,^81^,^82^,^83^,^84^,^85^. The topographical FC patterns could be identified in a vast majority of the deaf (Fig. 2A), including at the single-subject level (Fig. 2B), albeit with some variability among the subjects. Furthermore, our analyses revealed no large-scale differences between the deaf and the normally-hearing controls in their topographical FC patterns in the temporal lobe auditory cortex, in both univariate and multivariate analyses (Fig. 1G,H). In fact, the two groups FC-patterns were significantly spatially correlated (see highly significant CCC analysis), had similar between-subject variability (Fig. 2C), and were not distinguishable in an independent clustering analysis (Fig. 2D), suggesting that at least the large-scale topographical patterns are indeed alike despite life-long auditory deprivation. Moreover, these patterns are found despite cross-modal plasticity evident in the same deaf subjects, both in our results (in the FC between the early auditory cortex seeds and areas outside the temporal lobe, such as the insula; Figure S8; Tables S1 and S2), and in an additional study, which showed visual cross-modal plasticity in the same deaf group^14^.

These findings add to a large literature dealing with the balance between development of proper auditory organization and plasticity in deafness. These studies reveal preservation of the gross anatomical connectivity patterns (mostly shown in animal studies) alongside extensive functional deficits and reorganization, depending on onset, duration and etiology of deafness (reviewed in refs 5,12). Generally, along with significant evidence for cross-modal plasticity in the connectivity pattern of the auditory cortex of deaf animals, thalamic and cortical auditory cortex connections are largely retained following perinatal deafness^26^,^28^,^29^,^30^. However, there is inconsistent evidence regarding the topographical mapping in deafened animals’ auditory cortex. In some studies, rudimentary or smeared tonotopic mapping in auditory cortex was found to be grossly maintained in A1 in congenital deaf cats^26^,^29^,^86^, whereas other studies found that long-standing and early-onset deafness (in neonatally deafened animals) diminishes tonotopic gradients^87^,^88^. In addition to this inconsistency in the animal literature, the human auditory cortex differs to some extent in its organization from that of other mammals due to the development of language (e.g.^31^). In humans, non-congenitally deaf cochlear implant patients (who have had auditory experience) show evidence for auditory core tonotopic organization^89^,^90^ (although not always:^91^), and there is also indirect evidence for tonotopic responses in the brainstem of congenitally deaf children^92^. However, to the best of our knowledge, no direct mapping of the tonotopic, or topographic, organization was conducted in the CI-naïve congenitally deaf human auditory cortex, let alone in its association regions, without any auditory sensory experience. On the other hand, there is evidence for plasticity in this population: there are clear changes in white matter and gray matter volumes and correlation in early auditory cortex^93^,^94^,^95^, and changes in functional connectivity of the auditory cortex^9^,^96^,^97^. Such changes tend to emphasize cross-modal compensatory plasticity, especially with regard to language. Functionally, it is known that cochlear implantation outcome in prelingually (congenitally) deaf children is optimal for early implantation, in the first years of life^2^,^3^,^98^, and that later implantation is far less beneficial for auditory processing skills^4^,^5^,^6^. Therefore, there is grounds for surprise at the extent of topographical organization found in a group of congenitally, profoundly deaf subjects, who use mostly sign-language, have poor oral language skills and do not have a history of efficient use of hearing aids or cochlear implantation. All of these are factors that supposedly interfere with auditory cortex proper organization and promote cross-modal plasticity. Indeed, a recent study in the same subject group found evidence that their auditory cortex contains decodable information about the location of visual stimuli^14^. Thus, the ability to find retained FC tonotopic organization even in this group suggests that indeed such organization patterns emerge without reliance on auditory sensory inputs during critical periods.

Our findings suggest that some aspects of the tonotopic connectivity organization do not crucially rely upon auditory experience-dependent developmental mechanisms. These connectivity patterns likely develop based on genetic blueprints and activity-dependent mechanisms, by which the primary sensory receptor inner hair cells (IHCs) in the mammalian cochlea spontaneously fire action potentials prior to hearing onset^99^,^100^,^101^,^102^,^103^,^104^. Therefore, many of the auditory projections are already largely present prior to hearing onset^105^. However, a final refinement of such connectivity is likely to be achieved only as a result of auditory experience^5^,^12^,^103^,^106^,^107^. These results resemble those found in the visual modality, where the large-scale functional connectivity topographical architecture (reflecting retinotopic organization) can be found even in congenital blindness^49^, likely based on prenatal spontaneous patterned retinal firing^108^,^109^,^110^,^111^,^112^, although visual experience is needed for the generation of fully functional vision^113^. Similarly to blindness^114^, deafness also seems to cause changes in connectivity of the auditory cortex to other regions, such as the limbic and frontal cortex^95^, while retaining the intra-cortical organization relatively intact, as can be seen in our findings. Thus, similarly to the state of the visual cortex in blindness^49^, the deaf auditory cortex in humans shows a complex pattern of both plasticity and retained organization^115^.

While the similarity seen here between the deaf and hearing subjects was reproduced across various analyses, including both univariate and multivariate analyses, it is quite plausible that it may break down to some extent in a higher-resolution mapping of tonotopic gradient peaks, which may be conducted in the future in ultra-high field strength scanners (e.g. 7T;^64^). Such methodologies may also be able to better discriminate subcortical topographic FC effects (Figure S2A,B) and their plasticity (Figure S2D). The value of high-resolution future studies may be especially important as the absence of group differences cannot serve as evidence for equivalence (despite the highly significant spatial similarity between the maps in the CCC analysis and additional supporting analyses). Thus, a stronger power design may yet reveal significant differences between the groups also in the fine-detailed topographical FC within the auditory cortex (or subcortical structures), which was not detectable by the current methods. Moreover, it was recently discovered that tonotopic preferences even in the hearing population exist only at a relatively global scale as compared to the local cellular level^37^,^116^,^117^, and thus may not reflect directly the factors harmed by long-term or congenital deafness in deciphering auditory information. An additional factor which needs to be assessed is to what extent the intact resting-state FC observed here also represents the dynamic connectivity in the deaf brain, as resting-state FC and dynamic, state- and task-based connectivity show some differences^44^,^45^,^118^,^119^,^120^. It may be that the intra-auditory FC patterns manifest less during everyday life, such that the auditory cortex dynamically shifts its connectivity to better connect to the visual cortex and language regions, therefore showing plasticity due to lifelong experience and supporting cross-modal compensatory plasticity in the deaf. However, our finding of the underlying FC organization suggests that even if this is the case, the auditory cortex tonotopic network organization can still be found, and, potentially, utilized (see below). Lastly, a fully informed mapping of the effect of deafness on auditory cortex organization would have to take into account not only functional data and properties (such as tonotopic mapping) in animals and humans, but also the various cytoarchitectonic and myeloarchitectonic mapping models^91^,^121^,^122^,^123^,^124^,^125^,^126^,^127^,^128^, as these are crucial for the full delineation of auditory area borders and determining the areal organization (e.g. as was recently done in hearing subjects;^38^). Still, the non-invasive use of fcMRI can be useful to delineate functional auditory regions, even without the reliance on auditory stimulation. In the deaf, this method can provide auditory cortex delineation which may be useful in investigating in more depth both the retention of function as well as the different cross-modal changes in the plastic associative auditory cortex (e.g. refs 5 and 21).

Can the current findings and method also be of clinical benefit? It was recently shown that measures of cross-modal plasticity, as indexed by the degree to which the auditory cortex is recruited for non-auditory (mainly visual) processing^8^,^9^,^10^,^11^,^13^,^15^,^16^,^17^,^18^,^20^,^21^, may be used as a counter-indication for auditory restoration. Specifically, the degree of auditory cortex hypometabolism, generated by cross-modal reorganization^129^, (as measured by PET) before the operation was related to the amount of improvement in hearing capability after cochlear implantation, making temporal hypometabolism a possible prognosis factor for implantation^24^,^25^. Recently, more direct measures of cross-modal plasticity, auditory cortex activation for visual stimulation, were shown to negatively correlate with cochlear implantation outcome^23^,^130^. Therefore, assessing preoperatively the state of the auditory cortex in individual subjects may contribute to the decision to implant a CI, for example, beyond standard cochlear implant therapeutic usefulness ages^2^,^6^. This is especially true given that in many cases the cause of deafness or the onset of hearing deficit of any specific individual is not fully clear, and the auditory system organization greatly depends on these factors^5^,^12^. Thus, while it remains to be tested directly, it may be plausible that measures of intact auditory cortex organization such as the one tested here (which is reliable at the single-subject level, and yet rather variable and not present in all the subjects), especially at ultra-high field strength scanners might also have prognostic value.

Overall, our findings show that auditory cortex underlying connectivity large-scale patterns seem to develop properly and can be found despite auditory deprivation and cross-modal plasticity. This suggests that the balance of experience-independence and critical developmental periods should be further tested and revisited, for both the early and higher-order sensory areas, such as in the case of auditory language areas. These findings further stress the relevance of anatomical connectivity patterns and innate developmental constraints^131^ and their robustness in the face of plasticity.

## Methods

### Participants

15 deaf and 16 hearing subjects participated in the experiment. Participants in the hearing group were between the age of 18 and 22 (mean age = 20.1 years, 3 males), whereas participants in the congenitally deaf group were between 17 and 22 (mean age = 20.4 years, 2 males). Four deaf subjects and one hearing subject were excluded due to excessive head movement (>2 mm or spike-like motion >1 mm), leaving 11 deaf and 15 hearing subjects. All participants had normal or corrected-to-normal vision and had no history of neurological disorder. All experimental protocols were approved by the institutional review board of Beijing Normal University (BNU) Imaging Center for Brain Research in accordance with the Declaration of Helsinki, and all subjects gave written informed consent. The Chinese sign language was the primary language of all deaf individuals, and none could communicate using oral language in more than single words. All deaf subjects had hearing loss above 90 dB binaurally (frequencies tested ranged from 125 to 8000 Hz), and did not benefit from the use of hearing aids (used in the past by 5 of the subjects). The causes of deafness were genetic, pregnancy-related diseases or ototoxic medications, complications at childbirth, or unknown. See Table 1 for detailed characteristics of the deaf participants. The hearing participants reported no hearing impairment or knowledge of Chinese sign language.

### Functional Imaging

Images were acquired using a Siemens TRIO 3-T scanner at the Imaging Center for Brain Research, Beijing Normal University. The participants lay supine with their heads snugly fixed with foam pads to minimize head movement. The resting-state functional imaging data were comprised of 200 continuous EPI whole-brain functional volumes: 32 axial slices; 4 mm thickness; TR = 2000 ms; TE = 33 ms; FA = 73°; matrix size = 64 × 64; FOV = 200 × 200 mm; voxel size = 3.125 × 3.125 × 4 mm. During resting-state fMRI scanning, participants were instructed to close their eyes, keep still, and not think about anything systematically or fall asleep. T1-weighted anatomical images were acquired were acquired using a 3D MPRAGE sequence: 144 slices; 1.33 mm thickness; TR = 2530 ms; TE = 3.39 ms; inversion time = 1100 ms; FA = 7°; FOV = 256 × 256 mm; voxel size = 1.0 × 1.0 × 1.33 mm; matrix size = 256 × 256.

### fMRI preprocessing

Data analysis was performed using the Brain Voyager QX 2.8 software package (Brain Innovation, Maastricht, Netherlands) and complementary in-house preprocessing in MATLAB (MathWorks, Natick, MA) using standard preprocessing procedures. The first two images of each scan were excluded from the analysis due to non-steady state magnetization. Preprocessing of functional scans included the following steps: 3D motion correction, slice scan time correction, band pass filtering (0.01–0.1 Hz), regression of spurious signals from the ventricles and white matter regions defined using a grow-region function embedded in Brain Voyager on the individual level and spatial smoothing with a 4 mm full-width-at-half-maximum (FWHM) Gaussian kernel. The findings were replicated without spatial smoothing at the volume level, and in applying smoothing at the surface, cortical level, see detail below. Data in which head motion exceeded 2 mm in any given axis, or had spike-like motion of more than 1 mm in any direction (of four deaf subjects and one hearing subject) was excluded from further analysis. The remaining, used data did not differ between the groups in its motion parameters (maximum motion, p = 0.42, maximum motion range, p = 0.56). Data were then normalized to standard Talairach space^132^. T1-weighted anatomical images were used for surface reconstruction. Anatomical cortical reconstruction procedures included the segmentation of the white matter using a grow-region function embedded in Brain Voyager. The Talairach normalized cortical surface was then inflated and the obtained activation maps were superimposed onto it. Single subject activation maps are presented on the individual cortical sheets, thus preventing any potential misalignments. To further control for any distortions resulting from cortical inflation processes, the unsmoothed individual maps are also presented on individual cortical sheets in which the distances between the nodes on the surface were corrected using BrainVoyager (as done in^69^), such that they more accurately reflected their actual “native” distance in the original 3D volume (see Figure S4). To further validate that our group results in the deaf group were not in any way affected by the volumetric (Talairach based) spatial coregistration method used (Fig. 1), group analyses were also replicated in surface space. Surface-based alignment was conducted across the subjects according to their cortical curvature (sulci and gyri) patterns. The curvature alignment across the subjects was over 80% subject overlap for the Heschl’s gyrus anterior and posterior borders, showing that 9/11 participants data was fully aligned in this region, validating alignment success (see Figure S3A). As Heschl’s gyrus (regardless of its structure, single or duplicated;^65^) contains the primary auditory cortex central border between hA1 and R, this overlap verifies that the seed ROIs are sampled appropriately in the single subjects in surface space. The topographic patterns of the auditory cortex organization in the deaf were evident in this surface space control analysis with mild spatial smoothing at the volume level (4 mm FWHM; see Figure S3B,C), and without applying any spatial smoothing at the volume level, and instead using spatial smoothing at the surface level (4 vertex FWHM) following surface-based alignment (see Figure S5), to similar extents as in the main results. In addition to the univariate between-subject analyses, single subject analyses (Fig. 2; unsmoothed data Figure S4), multivariate analysis and spatial similarity analyses (see detail below) also support the high reproducibility of the findings and the appropriateness of the seeds.

### Defining a-priori tonotopic regions of interest

In order to define the primary auditory cortex seeds for fcMRI analysis we used data acquired from 10 normally hearing subjects (non-overlapping with the fcMRI hearing group) using a standard phase-encoded tonotopic mapping protocol^56^. This experiment included a rising logarithmic tone chirp spanning the range of 250–4,000 Hz in 18 seconds, followed by a 12 second baseline period, repeated 15 times. Stimulus intensity was set individually at levels between 86–89 dB SPL in order to optimize hearing on top of scanner noise. This data was analyzed using phase-encoded mapping, revealing the frequency preference of most of the core and high-order human auditory cortex (see Fig. 1A, adapted from^56^). For the purpose of the current investigation, seed regions-of-interest (ROIs) for fcMRI analyses were defined within the most significantly tone-selective region in the auditory cortex, corresponding to the auditory core (R(299) > 0.3, p < 0.0005 corrected for multiple comparisons;^56^). Seeds were taken from an area slightly posterior to the peak of preference for low frequency in left Heschl’s gyrus (corresponding to the primary auditory cortex) and from a more posterior area, in left Heschl’s sulcus, supposedly within A1 (which extends posteriorly from Heschl’s gyrus to Heschl’s sulcus;^65^), showing preference towards high frequency tones (see Fig. 1C). Both ROIs were of a similar size (590 mm^3^ and 533 mm^3^ for the LF and HF seeds respectively). The same strategy was used to define seeds in the right hemisphere for a supplementary analysis of interhemispheric topographical differences (Figure S9C).

### Analysis of functional connectivity

Functional connectivity was computed for a-priori defined seed regions-of -interest (ROIs) located in the primary auditory cortex (A1), selected according to tonal preference using a separate task-based tonotopic mapping external localizer from normally-hearing controls (see details above). Individual average time courses from each seed ROI were sampled and z-normalized at the individual level. Partial correlation analysis^49^,^57^,^58^ was performed in the following manner: for each seed, the time course was used as an individual predictor while regressing out the time course of the complementary auditory seed component (using it as a nuisance regressor). This was conducted to discard the shared variance and remain solely with the unique variance attributed to the first seed. This was computed both at the individual level, as well as in a group analysis (n = 11 for the deaf, n = 15 for the normally hearing) using a GLM in a hierarchical random effects (RFX) analysis^133^. This analysis resulted in a group map for each of the 2 ROIs. The two maps from these complementary seeds in A1 were overlaid to reveal the topographic FC pattern for each group separately (Fig. 1D,E). In order to investigate FC only in tonotopic areas, the results are presented in the areas showing significant cortical response to auditory stimulation in the external tonotopic experiment^56^ (p < 0.05, corrected for multiple comparisons using the Bonferroni correction). The minimum significance level of the findings, unless otherwise specified, was set to p < 0.05 corrected for multiple comparisons, using the spatial extent method based on the theory of Gaussian random fields^134^, a set-level statistical inference correction. This was done based on the Monte Carlo stimulation approach extended to 3D datasets using the threshold size plug-in for BrainVoyager QX, applied on the entire cortex. For better sensitivity of the multiple comparisons correction, group differences were also inspected within the auditory cortex temporal mask. For thalamus maps (Figure S2) a thalamic anatomical mask (containing the entire thalamus) was used for the multiple comparisons correction. The same analyses were conducted for the curvature alignment control analysis, after transforming the volumetric seed-ROIs to a cortical representation, with mild spatial smoothing done on the volume level (Figure S3) or with spatial smoothing applied only at the surface level (Figure S5), to control for any potential misalignment and smoothing effects. Group comparisons were conducted using ANOVA (Fig. 1F–H) modeling one within subject effect (FC to the two seeds based on tonotopic preferences) and one between subject effect (group). For ANOVA standard seed FC analysis was used in a combined GLM model for both seeds, without using partial correlations (without regressing out the time course of another seed). Group comparison was also replicated with partial correlation (Figure S8). Probability overlap across the individual subjects were computed from partial correlation individual maps, each at p < 0.05 corrected for multiple comparisons. The maps were overlaid and the percent of subjects showing activation at each voxel was calculated. To demonstrate that the topographic organization evident in the findings did not merely arise from spatial local correlations, we sampled the FC (t-values) from the HF seed of sequential spatial regions (10 vertices each) along the left superior temporal plain beginning in the HF seed itself and anteriorly (see sampling points at Figure S6A). T values were sampled for the deaf group FC from the left HF seed and from the right HF seed, to demonstrate that the same pattern of topographic FC (decrease and then increase in FC, in accordance with tonotopic organization) can be found across hemispheres regardless of anatomical distance from the seed (Figure S6B).

### Multivariate analysis

Analyses were performed using CoSMoMVPA, an MVPA toolbox in Matlab^135^. Searchlight pattern classification^136^ accuracies for discriminating unsmoothed neural FC patterns in the deaf and controls were computed using leave-one-out cross validation, that is, the classifier was trained using the data of 21 patterns and tested on its accuracy at classifying the unseen data from the remaining pattern (data was permuted such that group sizes matched and subjects were included, 1365 iterations). This procedure was carried out using all possible combinations of train and test patterns. The classification accuracies were averaged to give a mean accuracy score per test per neighborhood (sphere sized 4 mm radius). The average classification accuracy’s significance was computed by comparing it to that obtained from data with shuffled labels, permuted 2000 iterations. The resulting accuracy map was z-transformed and corrected for multiple comparisons using the spatial extent method, as described above. No significant effects were found using this analysis.

### Analyses of spatial similarity

In order to quantify the spatial similarity between the tonotopic gradients and FC maps, between the deaf and control group FC maps, and assess the discernibility of the two groups we computed several difference measures.

(1) Concordance correlation coefficients (CCC;^78^) were computed using in-house software written in MATLAB (MathWorks, Natick, MA). Concordance correlation coefficient values range from 1 (perfect spatial similarity) to −1 (perfect spatial dissimilarity). While CCC, similarly to Pearson’s linear correlation coefficient, tests for shared fluctuations in variance of two datasets, it also penalizes for differences in means between the two sets, thus serving as a more sensitive measure for group differences in both spatial patterns and overall FC. CCC values were computed between the tonotopic gradients preference (t-value difference between responses to HF and LF tones in the auditory external localizer; Fig. 1B) and FC map seed preference (t-value difference between FC of HF and LF seeds), to assess the similarity of the FC gradients with tonotopic maps. CCC was also calculated for each seed between the two group maps (e.g. between the low frequency seed map of the deaf and that of the hearing controls), and for the effect size (z-normalized effect size, HF-FC minus LF-FC). A mask of the auditory-responsive cortex (all areas showing auditory responses in the external auditory localizer, p < 0.05, corrected) was used to compute the similarity between the tonotopic gradients and FC maps and between the two group maps only for the relevant regions. The significance level was obtained using a permutation test (100,000 iterations) while randomly shuffling voxels from one group map and convolving the resulting map with a Gaussian kernel based on data smoothness estimation, to account for spatial autocorrelation. These values were then corrected for multiple comparisons using the Bonferroni correction.

(2) Testing subjects’ distribution of spatial FC patterns within and between the groups was conducted by applying a leave-one-out analyses of the z-normalized effect size (HF-FC minus LF-FC) within the auditory cortex (similarly applied in ref. 137). The averaged FC map of all hearing subjects except one was obtained. The correlation of the FC map of the left-out subject to the average was obtained and termed the pattern similarity score. This was repeated in a typical leave-one-out fashion. For the deaf subjects, the FC map for a given subject was correlated with each possible leave-one-out aggregate of the hearing or deaf subjects, and the average correlation across these partitions obtained.

(3) Hierarchical clustering analysis across subjects FC maps (z-normalized effect size, HF-FC minus LF-FC within the auditory cortex) was computed, using the linear shortest Euclidean distance of the correlation (1-r) between all subject pairs, creating a dendrogram of the distances across all the subjects (Fig. 2D). The distribution of the deaf and hearing subjects within the distance dendrogram suggests the deaf variability in tonotopically-informed FC maps is well within that of the hearing controls.

## Additional Information

**How to cite this article**: Striem-Amit, E. *et al*. Topographical functional connectivity patterns exist in the congenitally, prelingually deaf. *Sci. Rep*. **6**, 29375; doi: 10.1038/srep29375 (2016).

## Supplementary Material

Supplementary Information

## Figures and Tables

**Table 1:** Characteristics of the deaf participants.

Subject	Age	Gender	Hearing loss age	Deafness etiology	First language	Hearing aid history^1^	Hearing ability with aids^2^
**D1**	21	F	0	Hereditary deafness	Sign	0	
**D2**	21	F	0	Hereditary deafness	Sign	0	
**D3**	21	F	0	Maternal disease/drug side effect during pregnancy	Sign	0	
**D4**	20	F	0	Maternal disease/drug side effect during pregnancy	Sign	0	
**D5**	20	F	0	Maternal disease/drug side effect during pregnancy	Sign	0	
**D6**	19	F	0	Ototoxicity	Sign	0	
**D7**	19	F	0	Dystocia	Oral	1	1
**D8**	18	M	0	Maternal disease/drug side effect during pregnancy	Sign	1	1
**D9**	16	F	0	Maternal disease/drug side effect during pregnancy	Sign & oral	1	1
**D10**	20	F	0	Unknown	Sign & oral	1	1
**D11**	20	F	0	Ototoxicity	Sign	1	1

^1^Hearing aid history:

2. Using now

1. Used in the past

0. Never used

^2^Hearing ability with aid:

1. Hears something, but difficult to find out where it comes from

2. Distinguishes human voice, but can’t understand what people say

3. Hears and understands what people say well.
