# Supplementary material for: Topographical functional connectivity patterns exist in the congenitally, prelingually deaf
Source: Sci Rep. 2016 Jul 18;6:29375. doi: 10.1038/srep29375 (PMC4947901; doi:10.1038/srep29375)
Supplement: Supplementary Information [file srep29375-s1.pdf]

Supplementary Material

Topographical functional connectivity patterns exist in the congenitally, prelingually deaf

Ella Striem-Amit[1*], Jorge Almeida[2,3], Mario Belledonne[1], Quanjing Chen[4], Yuxing Fang[4], Zaizhu Han[4], Alfonso Caramazza[1,5] and Yanchao Bi[4*]

[1] Department of Psychology, Harvard University, Cambridge, MA 02138 USA;

[2] Faculty of Psychology and Educational Sciences, University of Coimbra, Coimbra 3001-802, Portugal;

[3] Proaction Laboratory, Faculty of Psychology and Educational Sciences, University of Coimbra, Coimbra 3001-802, Portugal;

[4] State Key Laboratory of Cognitive Neuroscience and Learning & IDG/McGovern Institute for Brain Research, Beijing Normal University, Beijing 100875, China;

[5] Center for Mind/Brain Sciences, University of Trento, 38068 Rovereto, Italy

* Corresponding authors: Ella Striem-Amit, Department of Psychology, Harvard University, Cambridge, MA 02138 USA, Email: striemamit@fas.harvard.edu;

Yanchao Bi, State Key Laboratory of Cognitive Neuroscience and Learning & IDG/McGovern Institute for Brain Research, Beijing Normal University, Beijing, 100875 China, Email: ybi@bnu.edu.cn.

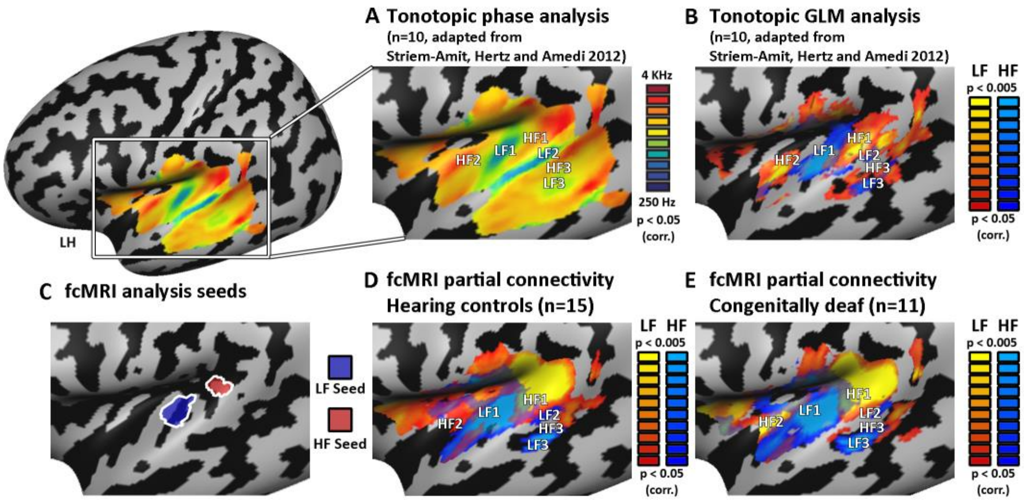

Figure S1: Marking of the tonotopic and comparable FC peaks

This figures depicts the tonotopic gradients and FC maps of the main results (**Figure 1**) while marking the peaks of tonotopic preference (**A,B**) and their parallel FC topographic peaks (**D, E** in the hearing and deaf). The core is characterized by peaks HF1, LF1 and HF2, while the extra-core structures include peaks LF2, HF3 and LF3. All these structure can be found to some extent in the FC organization in both groups, as well as in the individual subjects (**Figure 2**).

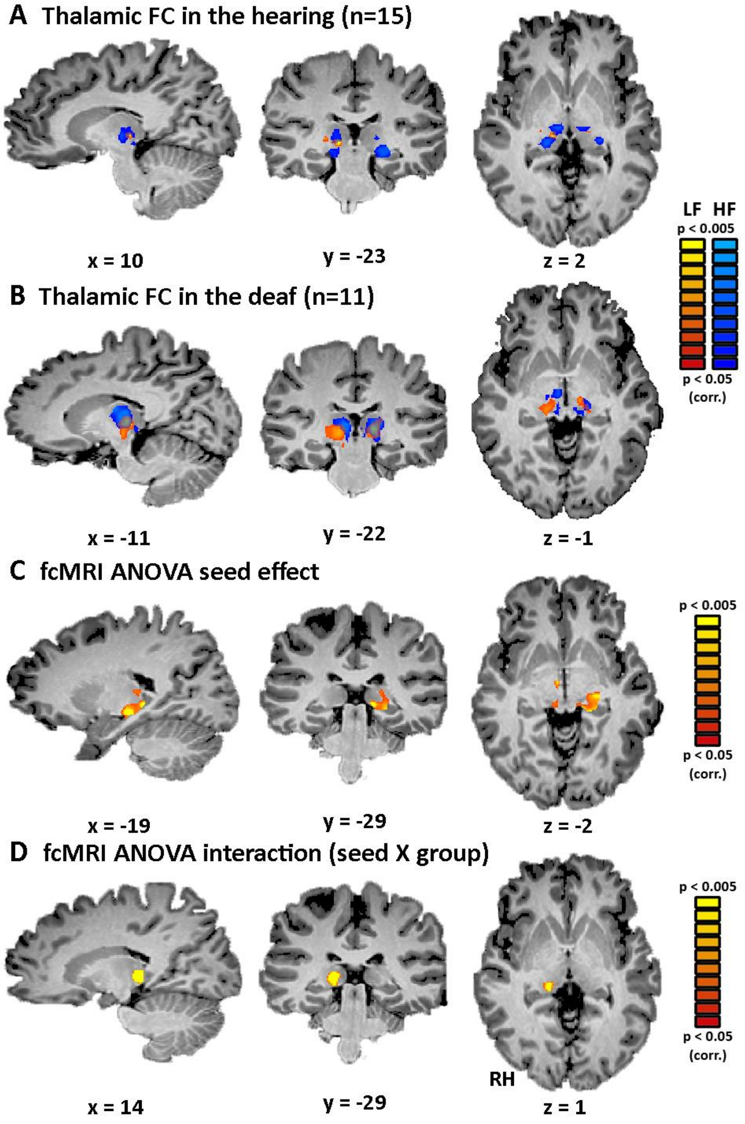

Figure S2: Thalamic FC from the auditory cortex in the hearing and deaf
fcMRI analysis (random effect GLM analysis; masked for display within the anatomical thalamus) shows auditory cortex FC in the MGN of the hearing (**A**) and deaf (**B**) groups, and a robust seed effect in the ANOVA (**C**). Although the current dataset may not be of sufficient spatial resolution to reveal group subcortical topographical patterns, the existence of FC back to the subcortical auditory network shows that such patterns may also be explored in the future using ultra-high field strength scanners (e.g. 7T; De Martino et al., 2013). Interestingly, group X seed interaction was also observed in the right MGN (**D**), suggesting potential plasticity in subcortical FC between the groups.

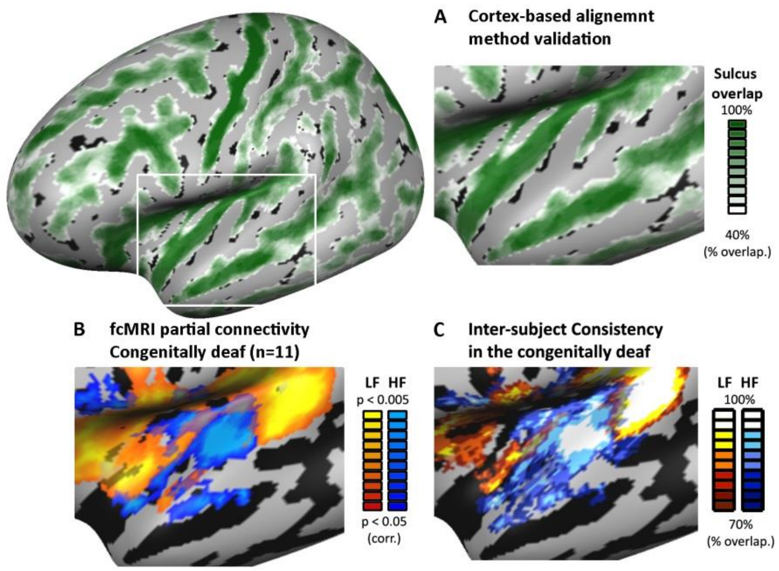

Figure S3: Cortex-based alignment fcMRI confirms topographic patterns in the deaf

A. The cortical surface depicts the result of the surface-based alignment. The overlap of the cortical curvature of all subjects with the MNI template used reaches over 80% in the anterior and posterior borders of Heschl's gyrus which mark the general location of the primary auditory cortex (the border between human A1 and R). This analysis validates the success of the cortical alignment procedure. For single subject analyses on the individual cortical sheets see **Figure 2**. **B-C.** The topographical tonotopic-like fcMRI patterns were also replicated using cortex-based surface alignment in both group analysis RFX GLM (**B**) and inter-subject consistency analysis (**C**), confirming the reliability of the findings**.**

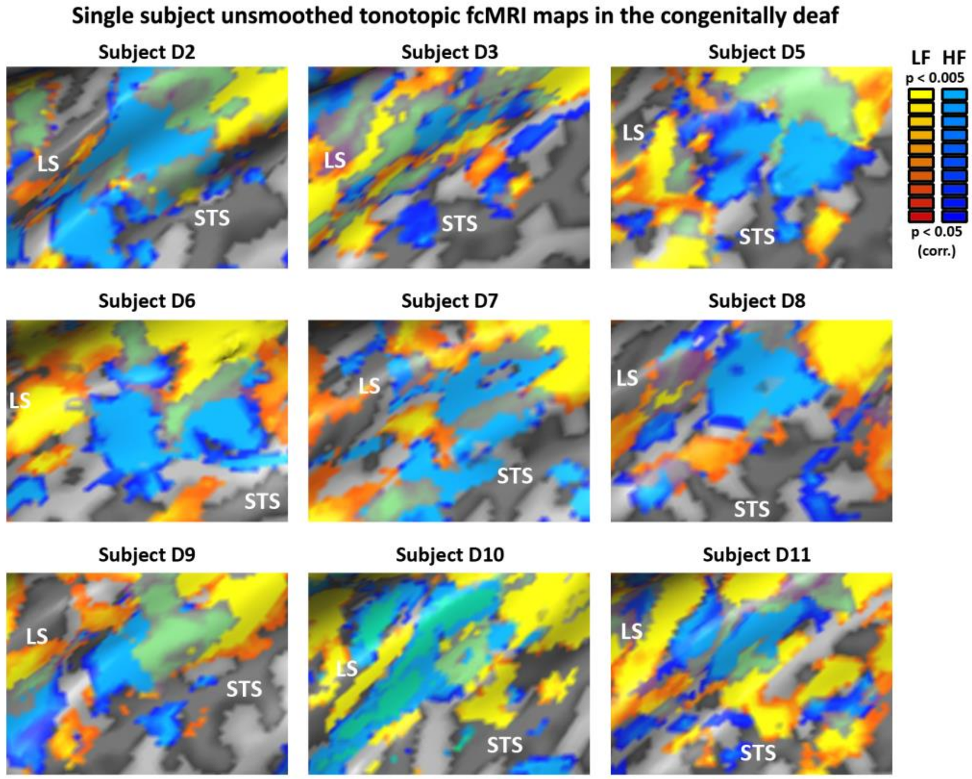

Figure S4: Reproducibility of tonotopic fcMRI maps in the single-subject unsmoothed data

In a comparable manner to shown in **Figure 2B**, the single subject data analyses were replicated using unsmoothed data. Topographical tonotopic-like fcMRI bands of the core and belt, including those of speech-sensitive regions, can be seen at the single subject level in many of the congenitally deaf individuals.

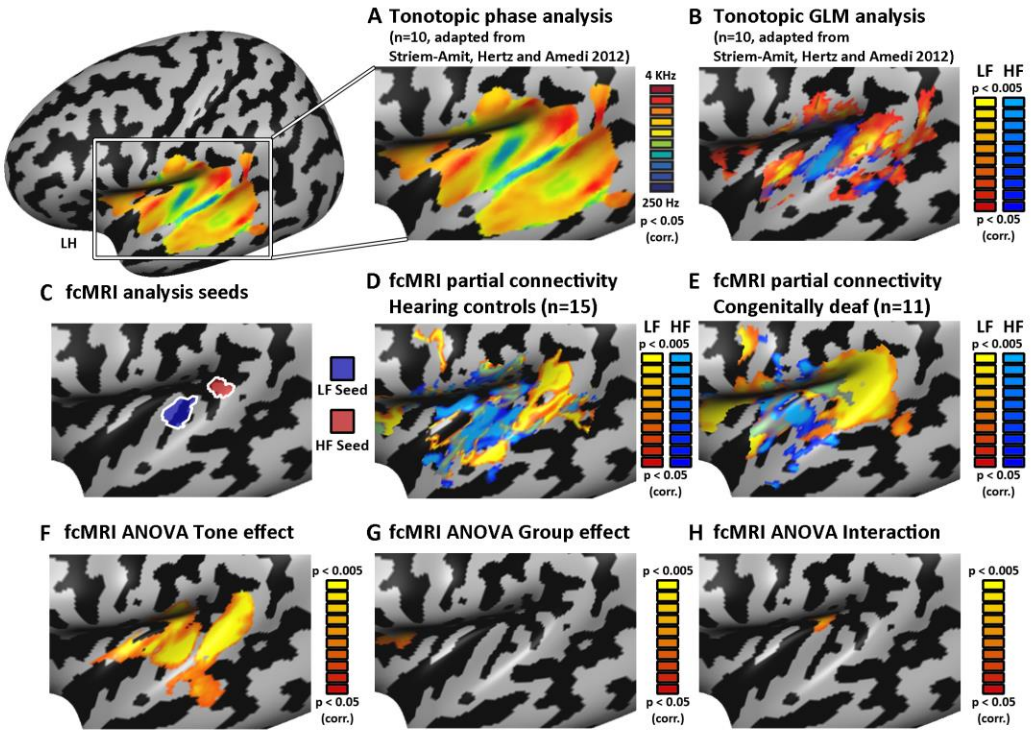

Figure S5: Cortex-based alignment fcMRI smoothed at the surface level confirms topographic patterns in the deaf
In a comparable manner to shown in **Figure 1**, the analyses were replicated using data not smoothed at the volume level, and smoothed mildly only after cortex based alignment (see alignment in **Figure S3A**). Tonotopically-informed FC is apparent to a similar extent in the hearing controls (**D**) and deaf (**E**), extending in both groups to the superior temporal gyrus and beyond it. Group analyses were also corrected for multiple comparisons at a smaller spatial extent, covering only the auditory temporal lobe, leading to a small cluster showing interaction between Group X Tone effects in the auditory cortex (**H**), possibly in the core or medial belt.

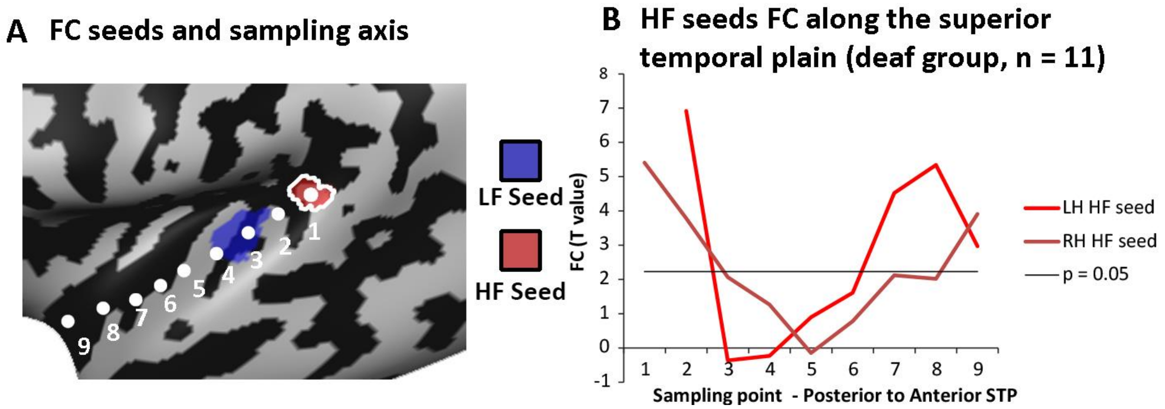

Figure S6: Local spatial correlations do not account for the topographic FC effects
To demonstrate that the topographic organization evident in the results did not merely arise from spatial local connections, we sampled the FC strength (t values) of nine sequential points (10 vertices each) along the left superior temporal plain beginning in the HF seed and anteriorly (points 1-9 in **A**; point #1 is enclosed in the seed itself). T values were sampled (**B**) for the deaf group for FC analysis from the left HF seed (bright red curve; does not contain point #1) demonstrating a decrease and then an increase in FC as sampling points are more anterior, a pattern resembling tonotopic topographic organization that cannot be explained only be local correlations. FC values were also sampled from these points from the FC to the right HF seed (dark red curve), demonstrating that the same pattern of topographic FC can be found across hemispheres regardless of anatomical distance from the seed.

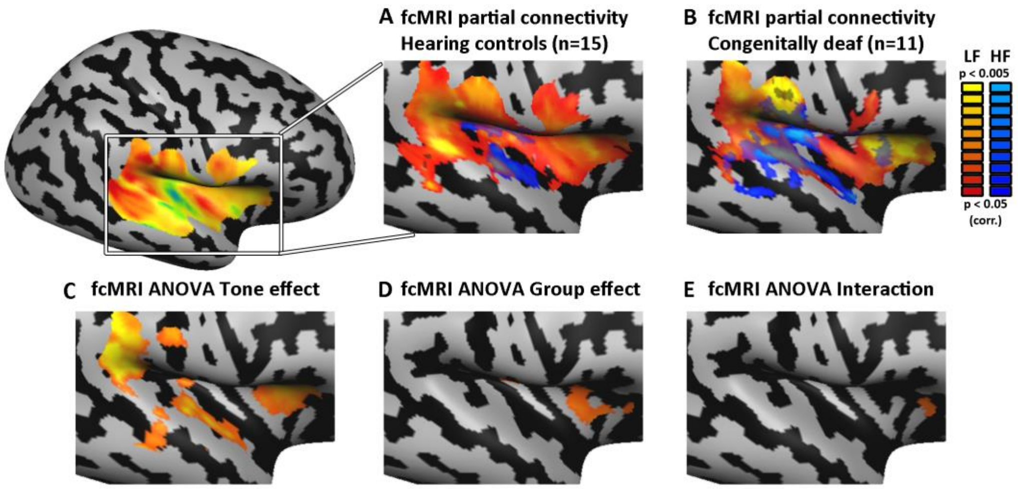

Figure S7: Right hemisphere topographic patterns emerging using left hemisphere fcMRI seeds
A. fcMRI analysis of the hearing control group (random effect GLM analysis) in the right auditory cortex from seeds in the left hemisphere (see seeds in **Figure 1C**) shows a more limited FC topographical organization than in the left hemisphere (same hemisphere seeding). This stands in contrast to the more complete topographical organization in the right hemisphere that be revealed using RH seeds (see **Figure S9**). Thus, while topographical patterns can be found in both hemispheres, it may be that tonotopy is a less important feature for interhemispheric cross-talk.
B. fcMRI analysis (random effect GLM analysis) of topographical organization in the right hemisphere auditory cortex of the congenitally deaf group using LH seeds is also less well-defined than within-hemispheric patterns (compare to that derived from RH seeds in **Figure S9**). This interhemispheric topographic-FC disconnection is similar to that observed in the hearing controls.
C-E. Analysis of variance (ANOVA) of the two groups shows that a smaller part of the right temporal auditory cortex of both groups is selective for one seed over the other (**C**, seed/tone effect), however, it shows no significant group effect (**D**) or group X seed interaction (**E**), which are only observed in the anterior insula (see full detail in **Tables S1,2**). This suggests that although FC topographical patterns are weaker across hemisphere, they appear similar in both groups and are not significantly affected by auditory deprivation.

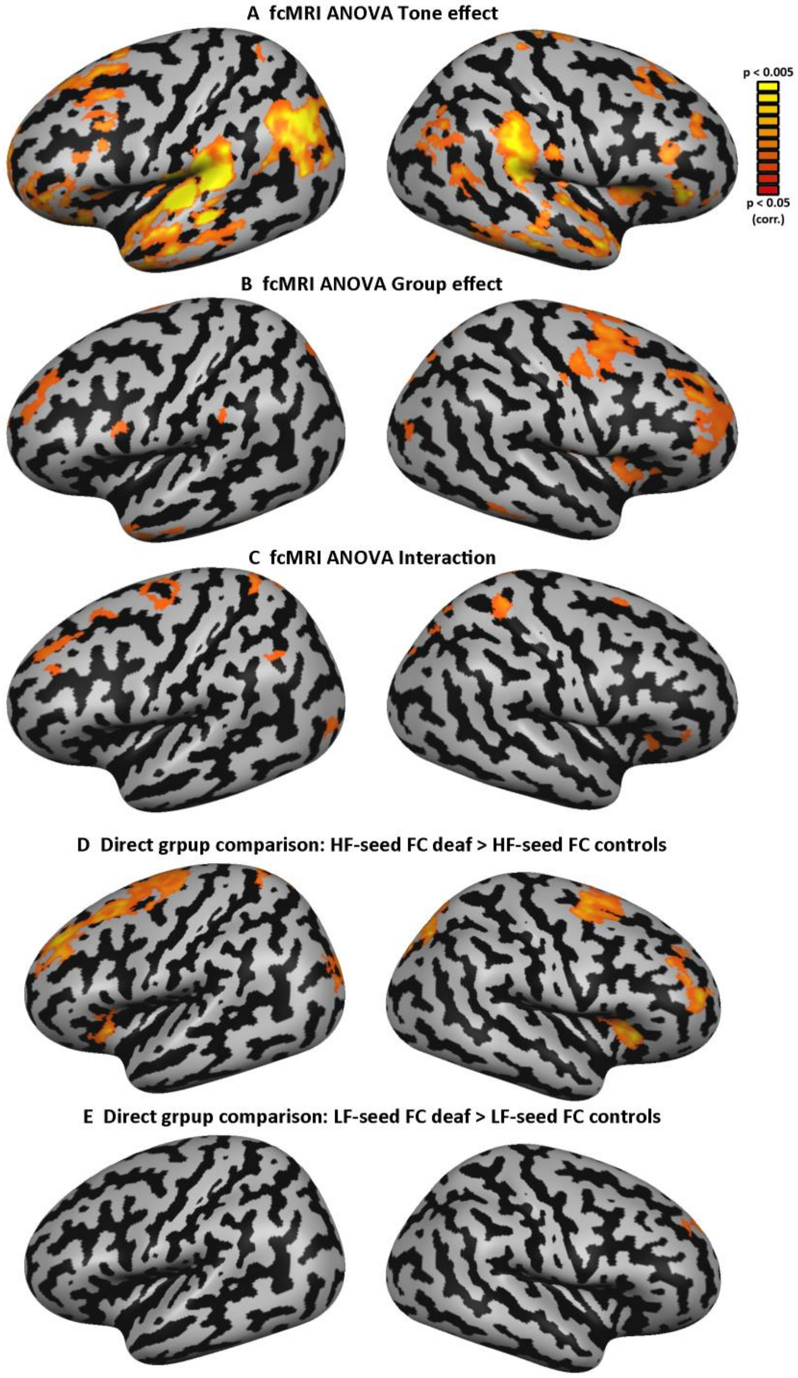

Figure S8: differential fcMRI patterns between the groups beyond the auditory cortex

A. ANOVA seed effect displays all cortical regions that show significant preference ($p < 0.05$ corrected for multiple comparisons using the spatial extent method) for seed ROI across both groups, including most of the left auditory cortex (and MGN; **Figure S2**), but also several regions in the bilateral parietal, frontal and temporal lobes. Most of which are likely parts of the auditory streams and multisensory integration areas also known from functional auditory task-based experiments (Romanski et al., 1999, Lomber and Malhotra, 2008, Rauschecker, 1998, Kaas and Hackett, 2000, Rauschecker and Tian, 2000).

B. ANOVA group effect shows regions whose FC to the auditory cortex differs between the deaf and the hearing. These do not include any temporal auditory cortex clusters, but rather mostly regions in the frontal lobe and anterior right insula. For a full table of peaks see **Table S1**.

C. The interaction between seed preference and group also does not show temporal lobe effects. For full detail see **Table S2** and **Figure S2**. Both **B** and **C** suggest that the FC topographical organization in the temporal auditory cortex does not change as result of congenital deafness.

D, E. A direct group comparison was computed with partial connectivity analysis for every seed-ROI separately, in the contrast deaf> control, replicating the ANOVA effects.

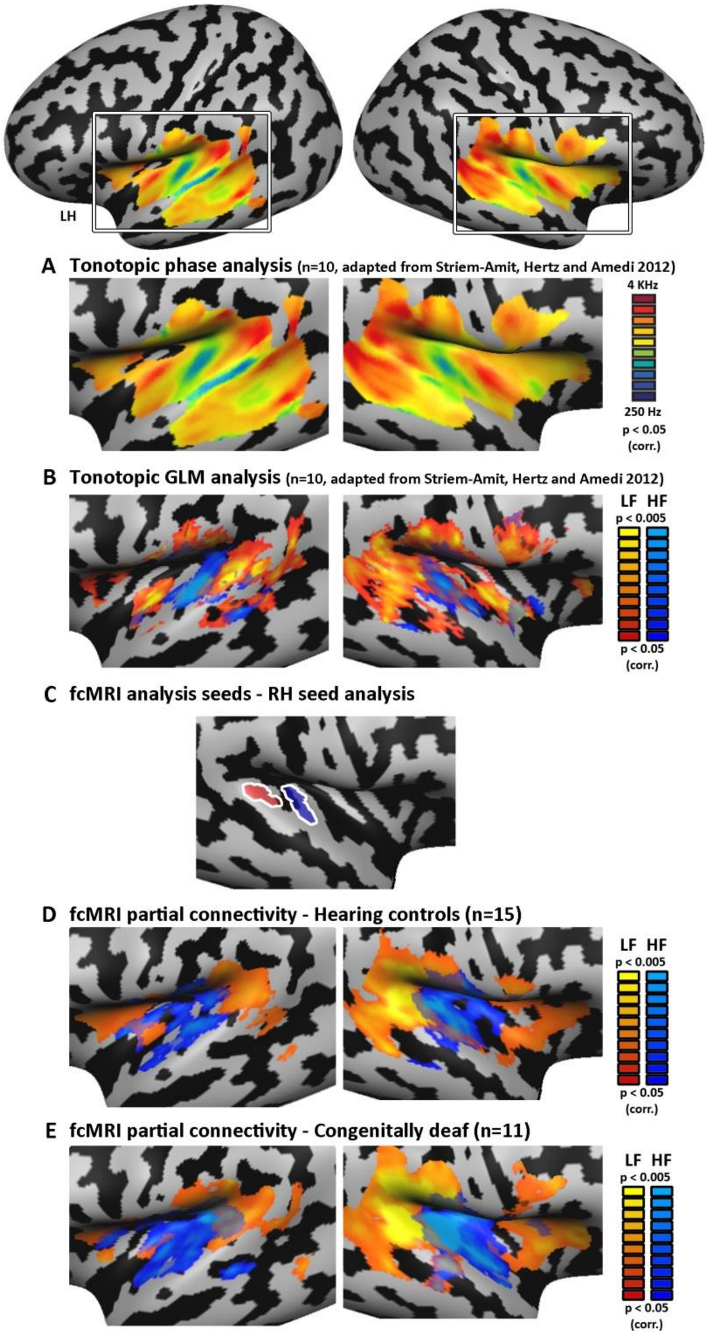

Figure S9: Topographical patterns emerging using RH fcMRI seeds

In a comparable manner to shown in **Figure 1**, using right hemisphere seed ROIs (**C**) for fcMRI analysis, topographical patterns resembling tonotopic organization (**A,B**) can be found in the right hemisphere both in the hearing controls (**D**) as well as in the congenitally deaf (**E**). As in the case of inspecting fcMRI patterns from left seeds in the right hemisphere, contralateral topographical pattern (in this case, in the left hemisphere) is more limited in both groups, and is observed mostly in the core gradients.

Table S1: Group differences in auditory cortex FC: increased FC in the deaf

Region		x	y	z
**LH**	Cingulate Gyrus	-6	17	34
**LH**	Cingulate Gyrus	-3	-10	43
**RH**	Cingulate Gyrus	9	-1	46
**RH**	Cingulate Gyrus	6	20	37
**RH**	Cuneus	12	-70	28
**RH**	Parahippocampal Gyrus	18	-46	7
**RH**	Middle Frontal Gyrus	30	-7	46
**RH**	Medial Frontal Gyrus	9	5	64
**RH**	Superior Frontal Gyrus	30	38	31

Table S2: Interaction effect – Group X Seed preference

Region	x	y	z
**RH Thalamus**	15	-25	4
**RH Lingual Gyrus**	9	-79	1
**RH Cuneus**	6	-70	7
**RH Cuneus**	21	-76	28
**RH Cuneus**	6	-61	4
**LH Lingual Gyrus**	-9	-61	1
**RH Precuneus**	18	-64	31
**RH Thalamus**	12	-34	-2
**LH Lingual Gyrus**	-6	-76	-2
**LH Cuneus**	0	-85	13
**LH Cuneus**	-12	-88	22
**RH Lingual Gyrus**	9	-82	-14
**LH Middle Occipital Gyrus**	-33	-67	4
**LH Lingual Gyrus**	-12	-94	-8
**LH Lingual Gyrus**	-6	-82	-17
**RH MidbrainRed Nucleus**	3	-22	-17
**LH Middle Temporal Gyrus**	-33	-64	16
**LH Parahippocampal Gyrus**	-21	-58	-11
**RH Superior Parietal Lobule**	24	-61	49
**RH Cuneus**	9	-73	34
**LH Superior Temporal Gyrus**	-45	-46	7
**LH Cuneus**	-21	-76	13
**LH Precuneus**	-24	-67	28
**LH Cuneus**	-18	-79	37
**RH Medial Frontal Gyrus**	6	-25	73
**RH Paracentral Lobule**	12	-40	67
**RH Paracentral Lobule**	21	-34	52
**RH Precuneus**	9	-55	64

**RH Precentral Gyrus**	18	-25	67
**RH Sub-Gyral**	24	-37	64
**LH Cingulate Gyrus**	-18	-25	46
**LH Medial Frontal Gyrus**	-9	-19	55
**LH Precuneus**	-9	-52	58
**LH Sub-Gyral**	-21	-43	61
**LH Postcentral Gyrus**	-12	-34	64
**LH Insula**	-39	-16	22
**LH Precentral Gyrus**	-21	-13	73
**LH Precentral Gyrus**	-30	-13	64
**LH Middle Frontal Gyrus**	-27	26	31
**RH Sub-Gyral**	9	-31	52
**LH Anterior Cingulate**	-6	11	25
**LH Cingulate Gyrus**	-15	20	28
**LH Sub-Gyral**	-18	-4	55
**LH Middle Frontal Gyrus**	-24	11	46
**LH Precentral Gyrus**	-42	-16	61
**LH Middle Frontal Gyrus**	-27	-10	46
**LH Middle Frontal Gyrus**	-21	8	61
**LH Anterior Cingulate**	-15	32	25
